# Machine learning detects altered spatial navigation features in outdoor behaviour of Alzheimer’s disease patients

**DOI:** 10.1038/s41598-022-06899-w

**Published:** 2022-02-24

**Authors:** Abhirup Ghosh, Vaisakh Puthusseryppady, Dennis Chan, Cecilia Mascolo, Michael Hornberger

**Affiliations:** 1 Department of Computer Science and Technology, University of Cambridge, Cambridge, UK; 2 Norwich Medical School, 2.04 Bob Champion Research and Education Building, University of East Anglia, Norwich, NR4 7TJ UK; 3 Department of Neurobiology and Behaviour, University of California Irvine, Irvine, USA; 4 Institute of Cognitive Neuroscience, University College London, London, UK

**Keywords:** Diagnostic markers, Alzheimer's disease, Scientific data

## Abstract

Impairment of navigation is one of the earliest symptoms of Alzheimer’s disease (AD), but to date studies have involved proxy tests of navigation rather than studies of real life behaviour. Here we use GPS tracking to measure ecological outdoor behaviour in AD. The aim was to use data-driven machine learning approaches to explore spatial metrics within real life navigational traces that discriminate AD patients from controls. 15 AD patients and 18 controls underwent tracking of their outdoor navigation over two weeks. Three kinds of spatiotemporal features of segments were extracted, characterising the mobility domain (entropy, segment similarity, distance from home), spatial shape (total turning angle, segment complexity), and temporal characteristics (stop duration). Patients significantly differed from controls on entropy (p-value 0.008), segment similarity (p-value ${10}^{-7}$), and distance from home (p-value ${10}^{-14}$). Graph-based analyses yielded preliminary data indicating that topological features assessing the connectivity of visited locations may also differentiate patients from controls. In conclusion, our results show that specific outdoor navigation features discriminate AD patients from controls, which has significant implication for future AD diagnostics, outcome measures and interventions. Furthermore, this work illustrates how wearables-based sensing of everyday behaviour may be used to deliver ecologically-valid digital biomarkers of AD pathophysiology.

## Introduction

Spatial navigation symptoms in Alzheimer’s disease (AD), such as disorientation or even getting lost, are often overshadowed by the predominant episodic memory problems. However, spatial navigation symptoms have much more significant implications than purely memory problems. In particular, spatial navigation has been reported to be one of the first cognitive abilities to be affected in AD, which occurs as a result of the disease pathology appearing early in structures which form part of the brain’s navigation centre (i.e., medial temporal lobe and parietal structures)^1^. Indeed, studies have suggested that changes to spatial navigation abilities could potentially represent a sensitive and specific cognitive marker for AD^2,3^. These studies have mainly investigated how individuals use the two main navigation strategies (i.e., egocentric/body-based and allocentric/map-based) to move in virtual reality (VR) environments^4^ , and their findings suggest that suboptimal performance on allocentric navigation tasks can not only help identify individuals that are in the preclinical stages of AD but also in predicting clinical progression of the disease.

Despite these strong findings for laboratory or clinic-based spatial navigation testing, there is much less evidence on how spatial navigation performance affects real-world navigation performance in AD. Most previous studies on real-world spatial navigation in AD, are based on anecdotal or single case evidence with little systematic evidence on the ecological impact of spatial navigation symptomatology. Exceptions are studies investigating getting lost events in AD, which studied the antecedents or consequences of such events but with little regard of how spatial navigation symptomatology might have contributed to those events^5^. We have previously published data investigating such events as well and trying to relate them to spatial navigation features, such as landmark density or road networks^6,7^. However, much less is known as to the everyday navigation patterns in AD and how the pervasive spatial navigation changes in the disease affect those patterns. This might allow to identify changes before AD patients get lost and mitigate the risks of such events occurring.

The current study addresses this issue exploring the real-life outdoor navigation patterns of community dwelling AD patients and age, gender matched healthy controls in real-world environments using GPS trajectory data. Given the novelty of this dataset, the data analysis was undertaken using two complementary approaches. First, we used a data-driven machine learning approach, where we specifically aim to investigate (a) how the outdoor navigation patterns of AD patients differ from controls with respect to various selected features and (b) if we can correctly classify all participants as being either AD patients or healthy controls, based solely on a combination of these selected features. We hypothesise that the outdoor navigation patterns of AD patients will differ from that of the controls on certain selected features, as results from previous studies have shown that AD patients have distinct outdoor navigation patterns in the community^8–11^. We also hypothesise that our machine learning classifier will be able to identify the AD patients and controls based on solely these features of their GPS data. Second, we tested the a priori hypothesis that AD would be associated with changes in navigational patterns as modelled using graph-based analyses. The rationale for this approach is based on the extensive knowledge that the hippocampus within the medial temporal lobe is critically involved in maintaining an allocentric representation of the environment that is a fundamental component of navigation^12,13^, and more recent work indicating that this hippocampus-based cognitive map can be modelled in terms of graph metrics^14^. Given that hippocampal degeneration is a central feature of AD, our hypothesis-led prediction is that topological measures of outdoor navigation would differentiate AD patients from control participants.

## Methods

### Participant recruitment

Sixteen community-dwelling AD patients and eighteen age and gender-matched healthy controls were recruited for this study. Inclusion criteria for the study were: 50–80 years of age, residing at home, and if a patient, having a clinical diagnosis for AD and with a carer (relative/spouse) willing to assist in the study. Exclusion criteria were: a previous history of alcohol or substance abuse, presence of a psychiatric condition, any other significant medical condition that may be likely to affect participation in the study (head injury, loss of vision, mobility issues), and for patients, the presence of a comorbid neurological condition not related to AD.

All patients were clinically diagnosed with AD using the National Institute of Neurological and Communicative Disorders and Stroke and the Alzheimer’s Disease and Related Disorders Association (NINCDS/ADRDA) diagnostic criteria^15^.

### Experimental protocol

The study adhered to all relevant guidelines and regulations. Ethical approval for this study was granted by the Faculty of Medicine and Health Sciences Research Ethics Committee at the University of East Anglia (Ref. FMH2017/18-94), as well as the National Health Service Health Research Authority (project ID 205788; 16/LO/1366). All AD patients had capacity to consent and did so independently. All research was conducted in accordance with the relevant guidelines and regulations. All participants underwent an experimental protocol consisting of a cognitive screening session and two weeks GPS tracking.

### Cognitive screening session

The cognitive screening session was held in a quiet testing room on the university campus for the controls and for patients, in a quiet room in their own home. In this session, the background demographics of the participants were collected from their carers such as their age, gender, and level of education. In addition, the participants completed the Mini-Addenbrooke’s Cognitive Examination (Mini-ACE), which is a validated cognitive screening test for dementia^16^. Participant scores on the Mini-ACE enable us to gauge their general level of cognitive functioning (i.e., higher scores indicating higher cognitive functioning) as well as screen for dementia (i.e., scores ≤ 25/30).

### GPS tracking

Following cognitive screening, all participants underwent GPS tracking of their outdoor navigation (i.e., outside the home) patterns in the community for a 2-week period. The two-week timeframe was chosen to capture the participants’ outdoor navigation patterns over repeated weekdays/weekends. The entire data collection period, across all participants, lasted from November 2018–November 2019 (i.e., 12 months and 14 days).

All participants were provided with a GPS tracker (Trackershop Pro Pod 5). They were asked to wear the tracker (i.e., by placing it in their coat/trouser pockets) whenever they left their house during the tracking period, regardless of the mode of transport used and whether they were alone/accompanied. Participants were also provided with a navigation diary and were asked to record the date/time of each outing, and whether they were alone or accompanied during the outing.

GPS data for the first batch of 22 participants (13 controls, 9 patients) were recorded at a sampling frequency of 0.33 Hz (i.e., one sample every 3 s), while for the remaining 12 participants (5 controls, 7 patients), it was recorded at 0.20 Hz (i.e., one sample—every 5 s). The differences in sampling frequencies are due to the GPS Company changing the lowest sampling frequency (from 0.33 to 0.20 Hz) of the devices online, midway through data collection. The devices recorded the following variables for each location data point-date/time, address (street name), speed (miles per hour), battery level (percentage), distance travelled (miles), signal accuracy (percentage), and latitude/longitude coordinates.

One patient’s data had to be discarded from the analysis due to them having a faulty GPS tracker and subsequently, insufficient collected data. Therefore, the data analysis was conducted on a total of 15 AD patients and 18 controls.

### Participant demographics

There were no statistically significant differences between controls and patients for age or gender, however controls had a significantly higher number of years of education than the patients. Group differences were seen in the Mini-ACE score, with patients performing significantly worse than controls; the scores of all patients met the upper cut-off of ≤ 25/30, indicating significant cognitive impairment for patients (Table 1).

**Table 1:** Participant demographics.

	Controls (mean; SD)	Patients (mean; SD)	Significance
Total sample	18	15	–
Age	68.33 (7.53)	70.33 (6.86)	ns
Education (years)	15.44 (3.11)	12.80 (1.78)	*
Male	9	8	ns
Female	9	7	
Mini-ACE score	28.52 (1.50)	18.13 (5.64)	***

*ns* not significant.

*p < 0.05, ***p < 0.001.

### Data analysis

While many analytical and data driven mobility models exist in the literature^17–19^, they are not readily applicable in our case of trying to identify individuals with AD from their outdoor navigation patterns. This is because these models in principle study statistical behaviour consistent across a large population (e.g., scale free behaviour), and identifies participants that fall outside this distribution; however, our sample size is too small to establish such normative behaviour. Further models^20^ targeted specifically to walking traces also do not apply to our analysis as we consider data that is from a variety of transportation modes. Thus, in this work, we study novel features extracted from segments of the outdoor navigation traces. We use this approach following common practice in audio processing and activity recognition from sensing data^21^, whereby the extracted segments provide tangible units of movement from the continuous trajectories and make the problem tractable.

#### Extracting segments

**Figure 1:**
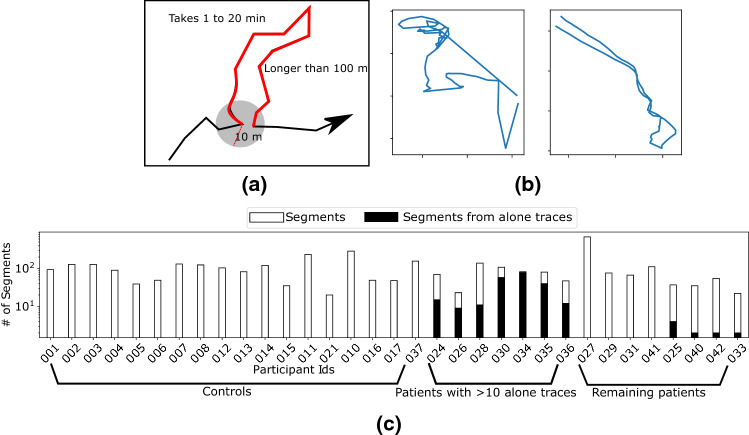
**(a)** A schematic to show an example segment extracted from the GPS trace. The red part is the segment we extract from the black trace. **(b)** Shows two example segments extracted from the dataset. **(c)** The number of segments extracted from the participants. The segments for the patients are marked according to whether they have moved with a caregiver or alone.

We define a segment as a sub-trajectory where the person returns to the same location (Fig. 1a,b). While returning to the same latitude and longitude is unrealistic, we consider a slack radius of 10 m for practical purposes. The duration of the segment needs to be between 1 and 20 min and the length of the sub-trajectory (i.e., the sum of lengths of the constituent linear pieces between location samples) needs to be at least 100 m long. The thresholds for the time interval and length are exploratory choices. The chosen upper threshold for the segment duration filters out long outings (e.g., daily trace), whilst the chosen lower threshold for segment duration and length filters out trivial short segments due to localization noise or being static at a place.

We extracted the segments in such a way that time intervals of no two segments overlap. The following computational method was used for segment extraction. For each location point $l$ on a participant’s trace, we find the locations within 10 m of $l$ using a KD-tree^22^. These locations constitute potential segment endpoints. If multiple segments exist with the same starting location, we consider the one with the maximum length. Note that the dataset contains all the movements carried out by the participants irrespective of the transportation modes used. Thus, the extracted segments also include different modes of transport.

#### Spatiotemporal features of the segments

**Figure 2:**
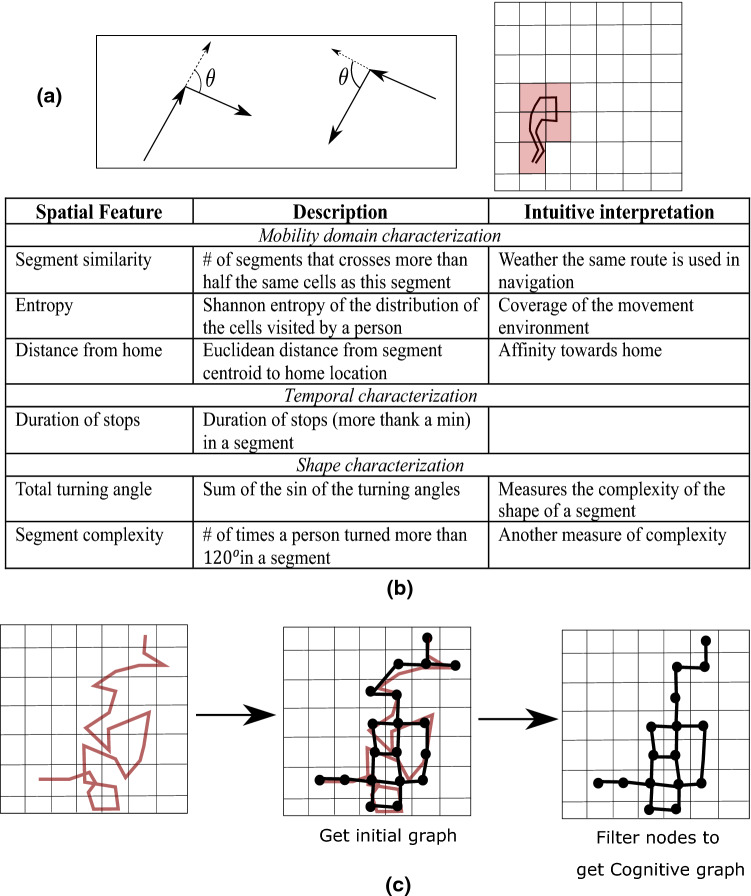
**(a)** Schematics explaining the spatial features: Left shows the angle between consecutive movement vectors and right shows a virtual grid and the red cells correspond to the cells crossed by the segment. **(b)** Summarizes the spatiotemporal features. **(c)** Overview of the process to create the cognitive graph from an individual’s mobility trajectory.

We investigate two types of spatial features from the segments characterising the mobility domain and the geometric shape of individual segments. Along with this we also study temporal properties of individual segments. As the number of segments varies across participants (Fig. 1c), we focus on the following features that are independent of the number of segments. The intuitive interpretation of the features is listed in Fig. 2b.

#### Mobility domain characterization

Features in this category aim to depict aggregate movement with all the segments from a participant. For these features we use coarser location resolution than GPS locations for computational efficiency and meaningful aggregation in terms of the visited location regions. Popular similarity measures for curves are expensive to compute^23^, in contrast, computing the common location regions crossed by a pair of segments is efficient. Here we present the results for an exploratory resolution of $100 \mathrm{m}\times 100 \mathrm{m}$, i.e., we lay a grid over the map and replace a location point with the index of the cell containing it.

##### Segment entropy

For each participant, we count the number of times a cell $c$ in the grid is visited (for example the red cells in Fig. 2a). Such normalized counts produce a probability distribution on the cells. We consider the entropy as $H= -\sum_{cell \,c}P\left(c\right)logP(c)$ where $P(c)$ denotes the probability mass at cell $c$. While considering the distribution, we discard the cells with zero values, which would otherwise introduce bias in the distribution. Our measure of entropy follows the definition based on information theory^24^. Intuitively, segment entropy is low when a participant’s movement has strong bias towards visiting a small number of locations within the grid, whereas the entropy is large for uniform distribution of visits to locations within the grid.

##### Segment similarity

The similarity between a pair of segments is defined as the Jaccard similarity of their intersecting cell IDs. We consider two segments as being similar if their Jaccard similarity is more than 0.5. Note that the Jaccard similarity between two sets A and B is calculated as $\frac{|A\cap B|}{|A\cup B|}$. The feature measures the fraction of segments from a participant that are similar to a segment.

Entropy and segment similarity have subtle differences. Entropy has a single value for a participant, and it measures how the segments are spatially distributed. For example, a person always following the same path will produce both low entropy and high segment similarity for segments corresponding to that path.

##### Distance from home

The dataset does not contain the home locations for the participants. Therefore, we estimate the home location of a participant as the centroid of the daily first and last locations. We then measure the Euclidean distance between home and the centroid of each segment.

#### Temporal characterization

*Duration of stops* measures the sequence of time durations a person remains static during a segment. We consider a person is static if the location does not change for at least a minute.

#### Spatial shape characterization

These features describe the geometric shape of the segments. They follow the popular measure for complexity of curves called tortuosity^25^ which is defined as proportional to the total curvature (or the turning angles) of a curve.

We first calculate the turning angles (Fig. 2a) along the segment and calculate two statistics as features. A segment is a sequence of vectors with location samples as the end points. At each location sample, ${x}_{i}$ we consider the first order turning angle, ${\theta }_{i}^{1}$ created by the vectors ending at ${v}_{i-1} ( <{x}_{i-1}, {x}_{i}>$) and starting at ${v}_{i}(<{x}_{i}, {x}_{i+1}>$) (Fig. 2a) and the second order angle, ${\theta }_{i}^{2}$ between the vectors, $(<{x}_{i-2}, {x}_{i}>,<{x}_{i}, {x}_{i+2}>)$.

##### Segment complexity

We first compute the average of the absolute first and second order turning angles at each location sample point, i.e., ${\theta }_{i}= \frac{{(|\theta }_{i}^{1}|+|{\theta }_{i}^{2}|)}{2}$. The complexity of a segment is the total number of ${\theta }_{i}$ that are more than the exploratory threshold of $12{0}^{\mathrm{o}}$. As we take the absolute values of the turning angles, complexity remains the same even with inverting the direction of travel. Further the averaging provides stability against noisy localization.

##### Total turning angle

It sums up the sine of the first order turning angles in a segment, i.e., ${\sum }_{i}{\mathrm{sin}(\theta }_{i}^{1}).$ The sine function captures the natural intuition of turning angles where the value turns negative beyond $18{0}^{\mathrm{o}}.$ This feature captures a different perspective of complexity of segments as it does not use any threshold (e.g., $12{0}^{\mathrm{o}}$).

While the angle turns are influenced by the underlying road network, large angle turns capture the ‘complexity’ of movements as such large turning angles are rare in a road network. Further, it must be mentioned that segments may indeed include movements denoted by walking; as walking traces can deviate from the road network, this feature provides an appropriate measure that quantifies movement beyond characterising road network. Example segments with their complexities are shown in Supplementary Fig. S2.

Supplementary material, S1 describes another feature to characterise the shape of a segment called, radius of gyration.

#### Graph-based features

In addition to the above, and in light of previous work indicating that navigation is structured as a “cognitive graph”^14^, we also modelled data in terms of graph metrics**.** For each participant a mobility network is constructed in the following way. First the location resolution is reduced using a grid in the same way as in the case of mobility domain characterization features. Each cell within the grid containing at least one location sample is considered a node in the graph. An edge connects two nodes if the cells contain consecutive locations in the trace. The resulting graph is then filtered by removing the nodes (cells) where the person stayed less than 5 min to ignore the places crossed in transit. While removing a node, we pairwise connect all its neighbours to maintain the same connectivity. Figure 2.c shows an example construction of a graph.

The graph-based features include the following centrality measures based on hop distances between nodes. *Closeness Centrality* of a node u is defined as the reciprocal of the sum of the shortest path distances to all other nodes from u. *Betweenness Centrality* of a node v is measured as the fraction of the shortest paths that pass-through v. *Degree centrality* of a node is the fraction of nodes it is connected to.

#### Participant features

We have considered two types of features, (i) where each participant has a single feature value, like entropy, and (ii) where each segment of a participant has a value like segment complexity. As the classification tools require a feature vector for everyone, we represent the type (ii) features using normalized histograms of the values from the segments.

### Ethics declarations

Signed informed consent was obtained from all participants before undertaking the experimental protocol. Ethical approval for this study was provided by the Faculty of Medicine and Health Sciences Research Ethics Committee at the University of East Anglia (FMH2017/18-123) as well as the National Health Service Health Research Authority (project ID 205788; 16/LO/1366).

## Results

We begin by presenting the statistical analysis of the spatiotemporal features leading to the classification of controls from patients. First, we consider the patients movements when they were alone. Next, we classify the segments from patients based on if they moved alone or accompanied. Finally, we present the results for cognitive graph-based features to separate controls from patients (without discarding the movements when accompanied).

### Spatiotemporal features

**Figure 3:**
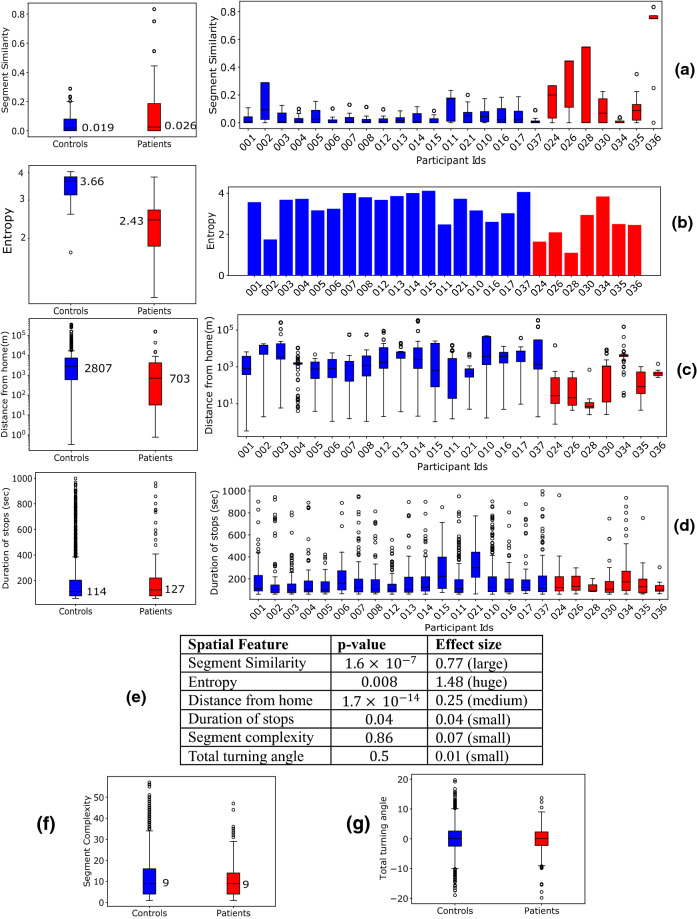
**(a–d)** Aggregate (left) and individual (right) distributions of feature values between control and patients. **(a–c)** are the features that produce best classification in Fig. 4. The boxes represent the region between 25 and 75 percentiles while the solid line inside the box denotes the median. All other values are shown as scatter points. **(e)** p-values (using KS-test) and effect size (Cohen’s d) between control and patients’ alone segments **(f,g)** Aggregate distributions for home distances and segment complexity.

Figure 3 studies spatiotemporal features from the controls and patients. Here, we only consider the patients’ segments when they moved alone; we selected the patients with more than five such segments—the dataset contains seven such patients. Figure 3a–d show the aggregate and individual distributions of segment similarity, entropy, distance from home, and duration of stops. P-values (Fig. 3e) are calculated between the distributions of the aggregated feature values from controls and patients using Kolmogorov–Smirnov (KS) test that measures the difference between the two distributions through the maximum separation of their cumulative distribution functions^26^ (See Supplementary Fig. S3). We choose KS test as it aligns with our choice of feature representation using histogram. We further report the effect sizes using Cohen’s d method^27,28^. Cohen’s d effect size measures the difference between the mean values of two populations normalized by a function of their sizes and variances.

For segment similarity, while the median values for the control and patients are close (Fig. 3a, left) (0.019 and 0.026 respectively), their distributions are significantly different with p-value being in the order of ${10}^{-7}$ and large effect size. Along with the aggregate distribution, the distribution of individuals (Fig. 3a, right) show that patients have higher segment similarity compared to controls.

For entropy, the population median values are 3.66 and 2.43 for controls and patients respectively (Fig. 3b, left), and the p-value is 0.008 with huge effect size. Both the aggregate and individual distributions show that the patients have lower entropy than the controls. The observation means that the places visited by patients are often spatially less diverse than the controls.

Further the patients move closer to their home locations compared to controls with median values for distance from home being 703 m and 2.8 km respectively (Fig. 3c). This is supported by the low p-value in the order of ${10}^{-14}$. The effect size is medium.

The separation between controls and patients is less clear for the duration of stops, segment complexity, and total turning angle (Fig. 3d,f,g)—the aggregate distributions produce p-values 0.4, 0.86, and 0.5 respectively. All of them have small effect sizes. However, the individual distributions for control and patients differ in the spread of the feature values (Fig. 3d).

### Patient classification based on spatiotemporal features

Here we study a binary classification between controls and patients using different combinations of the spatiotemporal features. Again, we used patients’ segments when they moved alone. A logistic regression classifier is used and the features represented as histograms with ten bins (Supplementary Fig S5 shows the performance for varying number of bins). We measure the efficacy of the classifier using sensitivity and specificity. Sensitivity is defined as the fraction of the patients among the participants predicted as patients by the classifier. Similarly, the specificity is defined as the fraction of the controls among the participants predicted as controls.

The classification is evaluated using leave one out strategy where all the participant’s data is used for training except one test participant and each participant is tested iteratively. We use stochastic gradient descent^29^ to minimize the logistic regression loss. All the parameters are set to the default values from the library except the number of iterations. As the optimization is stochastic, uses random initialization, and runs for a fixed number of steps (10,000), each experiment produces a slightly different result. Thus, each experiment is executed 50 times to estimate the uncertainty of the prediction which is represented as the standard deviations of the class probabilities in different runs.

**Figure 4:**
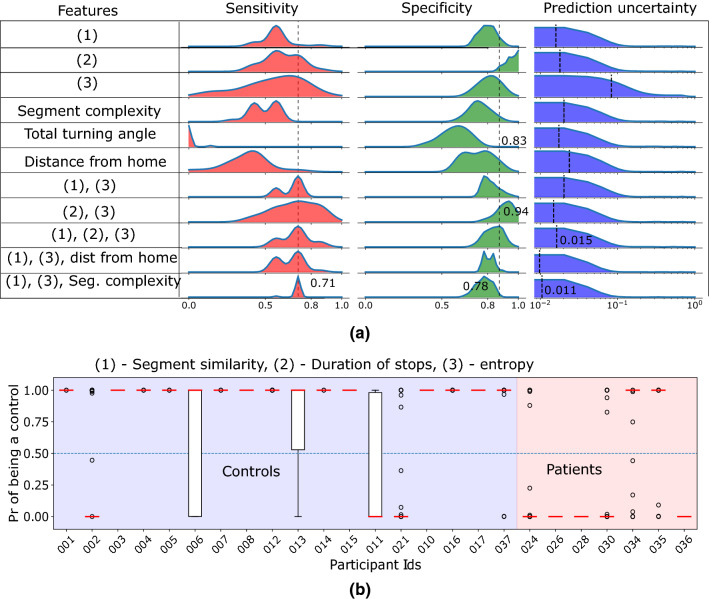
Classification results to separate the controls and patients while considering the segments where the patients moved alone. **(a)** Compare different combinations of the features by their sensitivity, specificity, and uncertainty (see the results for details) of the control probabilities. **(b)** Individual probability of being a control with the classification. An individual is predicted as a control if the probability is above 0.5 (the horizontal dotted line). The classifier uses segment similarity, duration of stops, and entropy as the features [last but one row in **(a)**]. The boxplots denote the distribution of the probability values produced by the classifier at different runs.

No feature achieves high sensitivity and specificity on its own, however results improve when combining a set of features (Fig. 4a). Entropy achieves best sensitivity of 0.85 as a singleton feature, however, it has large variance. Separately combining entropy with segment similarity and duration of stops improves the results significantly—both cases the median sensitivity is 0.71, and the specificity becomes 0.94 for the latter. However, the variance of the sensitivity remains large. Combining the segment similarity and entropy with either duration of stops, distance from home, or segment complexity reduce the variance and the uncertainty of prediction. All these combinations achieve the same median sensitivity of 0.71. Although the latter two achieve lower uncertainty and lower variance in sensitivity, the first combination achieves better specificity (median 0.83).

Figure 4b shows the individual classification probabilities when using the combination of segment similarity, duration of stops, and entropy as features. The best sensitivity then is 0.85 when only the participant 034 is wrongly classified. The median sensitivity is 0.71 where 035 is also wrongly classified along with 034. The large variance of the classification probability (longer bars in Fig. 4b) for 030, 034, and 035 denote that these participants lie close to the classification boundary in the feature space. All three participants 030, 034, and 035 had relatively high Mini ACE scores: 25, 23, and 24 respectively which correlate with the high uncertainty in classification. The classification achieves median specificity of 0.83 where 002 and 021 are wrongly classified.

### Classifying whether a patient was moving alone

Patients in the dataset have moved either alone or accompanied by their caregivers. In this section, we study a binary classification task to predict whether a given segment was produced when a patient moved alone. This task considers the same set of patients as the previous two sections and does not include controls. Here we aggregate the segments over the participants.

**Figure 5:**
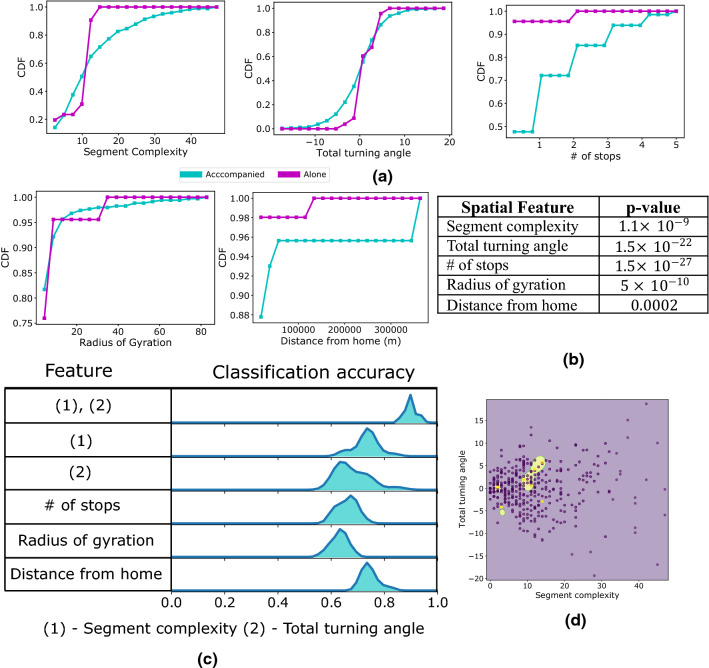
Classification of segments whether a patient moved alone versus accompanied. **(a)** The cumulative distributions for the spatiotemporal features. **(b)** p-value for the features derived from the KS test. **(c)** Classification accuracy while using different features, **(d)** classification boundary for the classifier when using (1), (2) together.

Figure 5 investigates different spatiotemporal features to characterise the geometric shape and the temporal behaviour of the segments. This uses the same set of features described in the methods, the only exception being the number of stops. All the features achieve low p-value (all below $${10}^{-3})$$ according to the KS test (Fig. 5b) with number stops having the lowest value in the order of $${10}^{-27}$$.

Alone segments have smaller segment complexity, total turning angle values and number of stops than the accompanied segments (Fig. 5a). They are also nearer to home than the accompanied ones.

The classification between alone and accompanied segments is studied in Fig. 5c. The experiment uses support vector machine with radial basis kernel. The experiment uses randomly chosen 20% of the data points as test samples and uses the rest for training. The experiment is repeated 20 times and at each run different accuracy is obtained due to randomness in the classifier initialization and test sample selection. None of the singleton features achieve accuracy beyond 0.8, however a combination of segment complexity and total turning angle produces median accuracy of 0.9. The classification boundary for this (Fig. 5d) is simple and the alone segments occupy a concise space in the feature space.

### Classification of patients and controls using graph-based features

Here we study the binary classification task with all segments from the patients (produced while alone or accompanied). This is more challenging than the patient classification considering only alone movements because when the patients are accompanied by their caregivers their mobility decisions may have been influenced. Thus, the features characterising the spatiotemporal shapes of the segments are meaningless. However, assuming broader mobility decisions are governed by the patients while the caregiver supports navigation, the spatial features characterising the mobility domain still are useful. These visiting patterns can further be characterized by the graph-based features.

**Figure 6:**
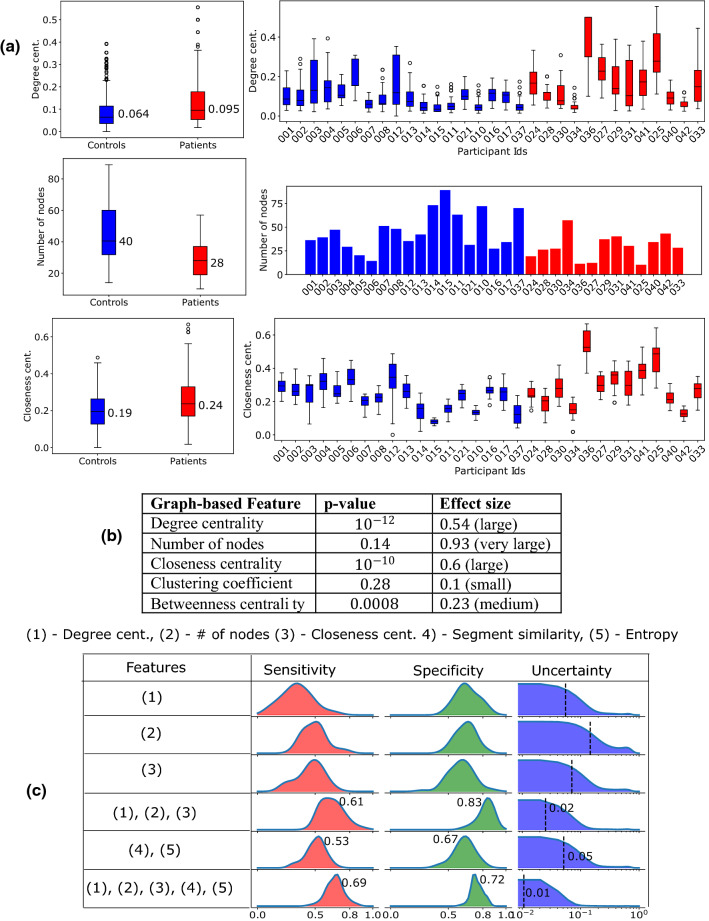
**(a)** Aggregate and individual feature distributions. **(b)** The p-values and effect sizes for the graph-based features. **(c)** Classification of controls from patients considering all segments from the patients (moving alone and accompanied). The best performing classifier (the last row) uses a combination of spatial and graph-based features.

Here we consider the participants that have at least ten nodes in their graphs—all controls along with 13 patients satisfy this criterion. Figure 6a shows the aggregate (left) and the individual (right) distributions for three features that achieve high classification results. The p-values and effect sizes are shown in Fig. 6b. The patients visit less number places as their graphs have lower number of nodes—median values are 40 and 28 respectively. Further the patients have nodes with higher closeness and degree centrality than the controls, i.e., they have a few fixed place(s) from which they go to other places.

Here we use the logistic regression and the same leave-one-out test methodology as used for classifying patients using spatiotemporal features.

None of the singleton graph-based features achieve good classification accuracy (Fig. 6c). The combination of the segment similarity and entropy also produce poor sensitivity (median 0.53) and specificity (median 0.67). While the combination of the graph-based features achieves the median specificity of 0.83 and median sensitivity of 0.61, it suffers from high uncertainty (mean value of 0.02). Finally combining the spatial features with the graph-based features produce median sensitivity of 0.69 and median specificity of 0.72. Further the mean uncertainty is also reduced to 0.01.

## Discussion

Taken together, outdoor navigation patterns of AD patients differed from that of controls, with respect to the spatial characteristics of the extracted trajectory segments and connectivity properties of the visited places, in line with our primary a priori hypothesis. In more detail, we found that when compared to controls, patients had a significantly lower segment entropy (i.e., more ordered), a higher segment similarity and moved less far away from their home location. Patients having more ordered and similar segments could potentially be explained by reports from previous studies that patients exhibit an increasing reliance on using familiar routes to navigate, potentially as a means to compensate for their declining navigation abilities^30–32^. Similarly, patients moving less distance from home suggests that they confine their outdoor navigation to very familiar locations, which may potentially represent a safeguarding mechanism to protect themselves from getting lost in the community as a result of their impairments in navigation^33^. It is as present unclear why patients have lower segment complexities than controls and this requires further investigation, however this could potentially be related to declines in mobility that are widely reported in AD patients, specifically in making turns^34,^^35^.

In addition to the above data-driven outcomes, we tested the a priori hypothesis that AD would be associated with disrupted navigational graphs, given previous work showing that hippocampus-based mapping of the environment could be measured as a cognitive graph^14^. However, limited availability of data from the relatively short evaluation window did not allow acquisition of sufficient base topological data points on nodes (places visited) and paths (routes taken between places visited) to construct robust graphs for effective analysis, especially for the case when the patients moved alone. Despite our exciting, initial results for modelling navigational data to classify controls from patients using graph-based analyses, future studies in large scale, longitudinal AD cohorts are required to explore the true potential of these techniques.

Regarding the results of the classification task, it is important to mention that the trajectory data is composed of movement both when the patients are alone and when they are accompanied by their carers. As the extent to which the carer may have influenced the outdoor navigation patterns of the patient (in moments when they were accompanied) is unclear, we first analysed a cleaner signal—when the patients moved alone. A combination of segment pairwise similarity, segment entropy and duration of stops produced the best classification result: a median sensitivity of 0.71 and specificity of 0.83.

The shape-based features produced the best results while classifying the movements when the patients moved alone from when they were accompanied. Accompanied segments show higher complexity in terms of both segment complexity and total turning angle features. This is likely due to patients requiring less navigation skills during an outing with their carer or other people and therefore, take more complex routes. These features achieve 90% classification accuracy, and it reinforces the intuitive idea that the navigation patterns are influenced by the carer. Further we explored classifying controls from patients without filtering out the traces when they moved accompanied. For this task, we explored the cognitive graph-based features and found that they improved the classification, yielding a median sensitivity of 0.69 and specificity of 0.72.

The geometric shape-based features, namely total turning angle and segment complexity do capture features beyond the noise in GPS localization (Supplementary Fig. S6). This is because as all the participants are given the same localization device, the calibration and the sampling rate remain consistent, this reduces the possibility of the complexity arising solely due to noise. Critically, it is difficult to apply popular localization noise removal methods here^36^. This is since the existing de-noising methods discard out-of-distribution location points considering them as localization noise, whereas in the context of characterising AD, we are looking for patterns outlier to control population. Previous studies had linked complex movement in indoor environment to cognitive impairment, in contrast, our results shows that the outdoor mobility differs from the indoor setting in an intricate way^37^.

Despite our promising results, there are some important limitations to our study that need to be mentioned. Our results show that the classification accuracy reduces when the data includes movements made by patients when they were accompanied by their carers. However, datasets generated by passive sensing will naturally contain a mixture of location traces when the tracked subjects were alone and accompanied. Though we show promising initial results in automated classification of alone/accompanied, such labelling, in general, is difficult to be performed in an automated way as part of the post-hoc analysis simply because the carer may have different level of influence on mobility. Therefore, a larger dataset comprised of more participants tracked for a longer period of time is necessary to provide insights into the robustness of the features. A larger dataset will also enable the usage of complex classification methods, for example deep learning based techniques, to improve accuracy. This might overcome some of the variances in the classification accuracy in the current data set. Moreover, small sample size may also have effect on the combination of features to predict group classification. Still, the effect sizes of the data shows that our results are robust, and it is encouraging that in even such a small sample outdoor navigation, patterns can be detected reliably. Clearly, future data collection is needed to replicate and extend our findings.

As location data are sensitive, its long-term usage for tracking has natural privacy implications. In spite of several efforts to anonymize location data^38,39^, it remains an open problem mainly because of the uniqueness of the spatiotemporal points in individual traces. However, the privacy risks can be lowered by deployment strategies. In a centralized learning paradigm, training needs the data from multiple participants to be accumulated in a single machine (such as the dataset we use in this study), and then the trained model can be deployed in a participant’s personal device in the wild. As the inference can be done at the device itself, the data from the users in the wild do not need to travel to the server and thus this preserves privacy. Moreover, following the recent advancement in machine learning, federated learning provides an alternate strategy to train the classifier in a distributed fashion without sharing the data to a central server^40^.

In conclusion, to the best of our knowledge, this is the first study to investigate whether one can identify AD patients solely based on GPS data of their outdoor navigation patterns in the community. Our results highlight the potential utility of real-world navigation patterns as an ecologically valid behavioural marker of AD. However, as the sample size in our data is small our results should be viewed as an initial proof of the concept. Knowledge gained from this study can inform the future study of navigation in AD, and in particular applying the metrics evaluated in this study to real world navigational data obtained from people in earlier stages of AD, when pathology manifests in the brain regions underpinning navigation, to determine the utility of this approach to detect AD prior to the onset of dementia. Further, it will inform future studies in patients with AD who are at risk of getting lost and one can develop algorithms based on real-world GPS data which calculate risk factors for AD patients getting lost.

## Supplementary Information

Supplementary Information.

## Data Availability

Due to privacy concerns for the GPS data, we do not make the data public, however we are happy to consider data requests on individual basis.
