# Supplementary material for: Machine learning detects altered spatial navigation features in outdoor behaviour of Alzheimer’s disease patients
Source: Sci Rep. 2022 Feb 24;12:3160. doi: 10.1038/s41598-022-06899-w (PMC8873255; doi:10.1038/s41598-022-06899-w)
Supplement: Supplementary file 1 — Supplementary Information. [file 41598_2022_6899_MOESM1_ESM.docx]

## Supplementary material

### S1 More spatial features.

*Radius of Gyration.* We compute the radius of gyration for a segment (S) about its centroid (C). The centroid of the segment is the mean of the location samples in the segment, $C=\frac{1}{|S|}\sum_{loc_{i}in S} loc_{i}$. The radius of gyration of a segment is computed as $r = \sqrt{\frac{1}{|S|}\sum_{loc_{i}in S} dist(loc_{i}, C)}$, where the $dist(. , .)$ measures the Euclidean distance.

Note that the value remains constant under rotation or translation of a segment. Intuitively, running around alike shaped parks produce similar radii of gyration independent of the location of the parks and the direction of the movement. However, the feature produces different radii if the movement is biased towards a certain region which changes the centroid (Fig S1).

Intuitively, it captures the shape of the segment in terms of distribution of time spent at its different parts.


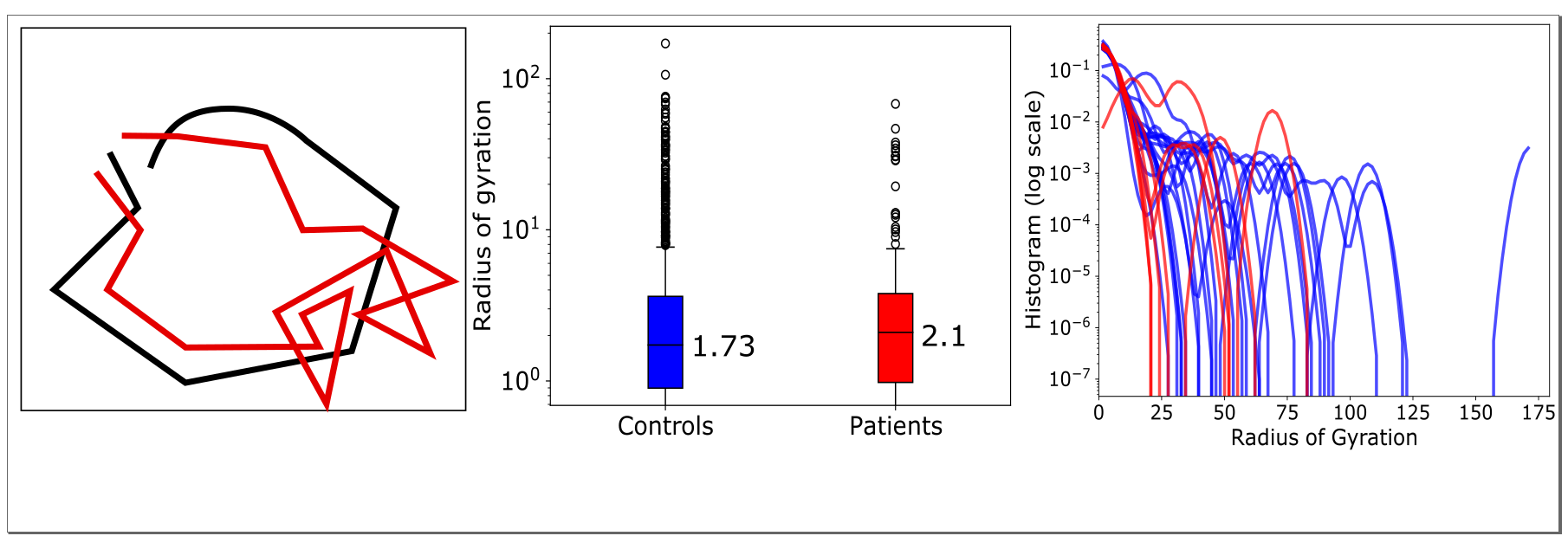


**Figure S1.** Radius of gyration can differentiate between the red and black traces as the centroid of the traces are different. While the median of the values in the control and the patient population are similar (middle box plot), their individual histograms differ (right).

*Speed.* The average speed in a segment is similar for both controls and the patients with medians 0.31 m/sec and 0.28 m/sec. Thus, we do not consider it in analysis.

### S2 Robustness of the features against localization noise.

Let us consider the localization noise producing locations outlier with respect to the neighbouring locations in the mobility trace. Below we argue that the features are robust to this kind of noise. As all the participants carried identical devices, thus localization noise due to location sensing calibration would remain consistent across participants.

Noise can increase the complexity of a segment by at most the number of outlier location points. However, if several outlier points are grouped together, then this is proportional to the number of such groups.

The effect of localization noise is also bounded in other spatial features. As outlier locations are randomly distributed in the space, they are unlikely to change the maximas of the spatial distribution of the segments. Thus, entropy remains similar under noise. Segment similarity is also robust to the noise for the same reason as a randomly picked outlier location can reduce the similarity value by a constant amount. The value of the radius of gyration for a segment also remains similar under outliers as the centroid is stable under outliers assuming a limited number of outliers.

### S3 Location privacy and on-device computation.

The system for detecting Alzheimer’s disease needs to track location continuously which is privacy invasive to the participants. Though a rigorous privacy argument remains elusive in this paper, none of our spatial and the cognitive graph-based features reveal actual locations in the traces. Exception being the turning angles that contain enough information to reconstruct the location coordinates of the trace.

This could lead to the following privacy friendly system design using on-device computation methods. Tracking devices can compute the feature values and transmit them to a central server at a certain interval (e.g., weekly). As the features are easy to compute, even resource constraint tracking devices can do this efficiently. Upon receiving the data, the central server can run the inference and update its model as required.

If the tracking devices choose to transmit the raw feature values for each segment (this would give the analyser more flexibility), there can be the following privacy implication. For example, suppose the adversary knows what the typical profile of feature values is when someone moves in a particular region (like a park), then finding such value in the published data can potentially reveal that the person went to the target region. We leave this problem for future research.

### S4 Robustness of the features against heterogeneity.

The number of segments by the patients and controls especially while considering the movements when alone are unbalanced. Thus, we do not consider the number of segments to be a feature for classification. And the spatial features we consider do not depend on the number of segments. For example, the entropy feature can reach maximum with only a small number of segments (with uniform random distribution among the visiting cells). Further the other spatial features measure a fraction with respect to the total number of segments instead of an absolute number. The same argument applies for the graph-based features as well.

### S5 Effect of segment length

In the experiments, we have chosen exploratory thresholds for creating the segments. Here we increase the duration threshold for creating a segment to 30 minutes instead of 20 minutes. This change will produce longer segments. Below we find that the entropy of the resultant segments is still different between the control and the patient population.


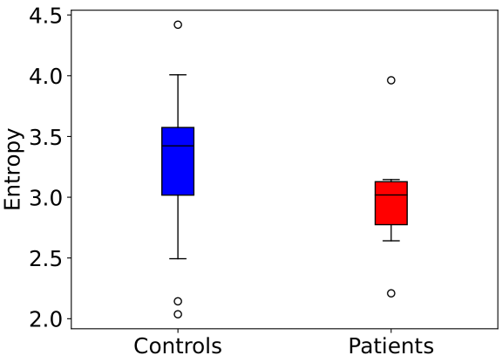


### Supplementary Figures


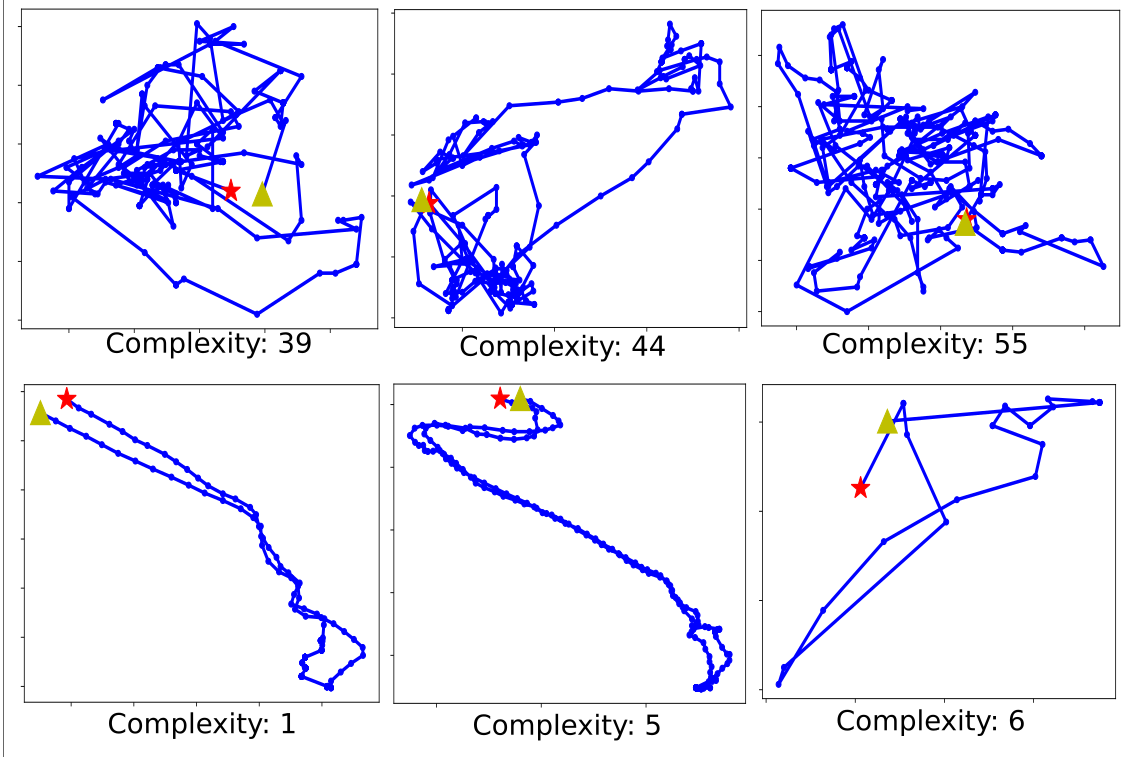


**Figure S2**. Example segments with their complexities. The star and the triangle denote the start and end points of the segment.


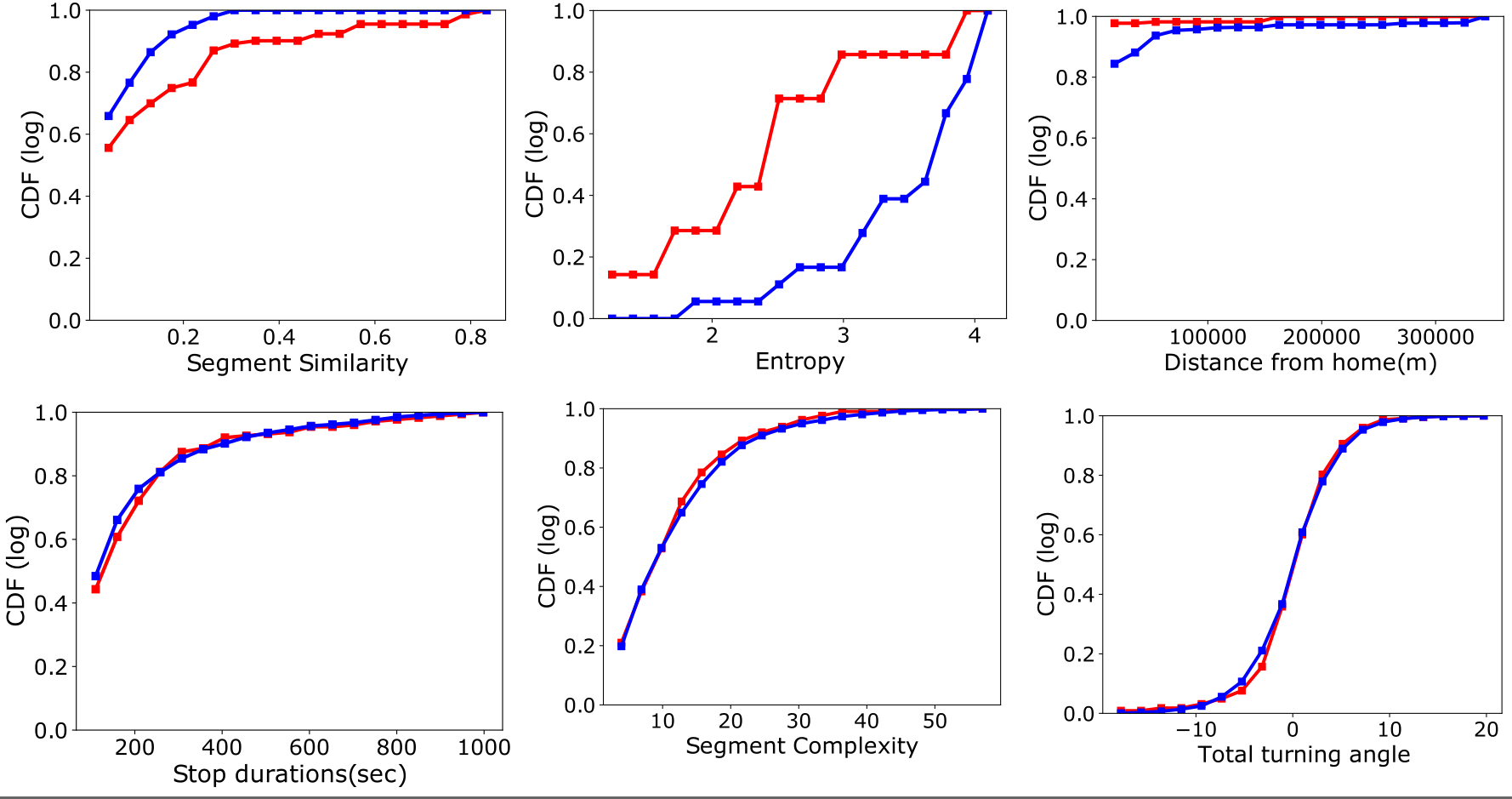


**Figure S3**. Cumulative distributions of the spatiotemporal features when considering the patients with alone segments. Patients have larger segment similarity, lower entropy, and lower distance from home. The differences in the other features are not visually detectable. Patients and controls are represented by red and blue lines respectively.


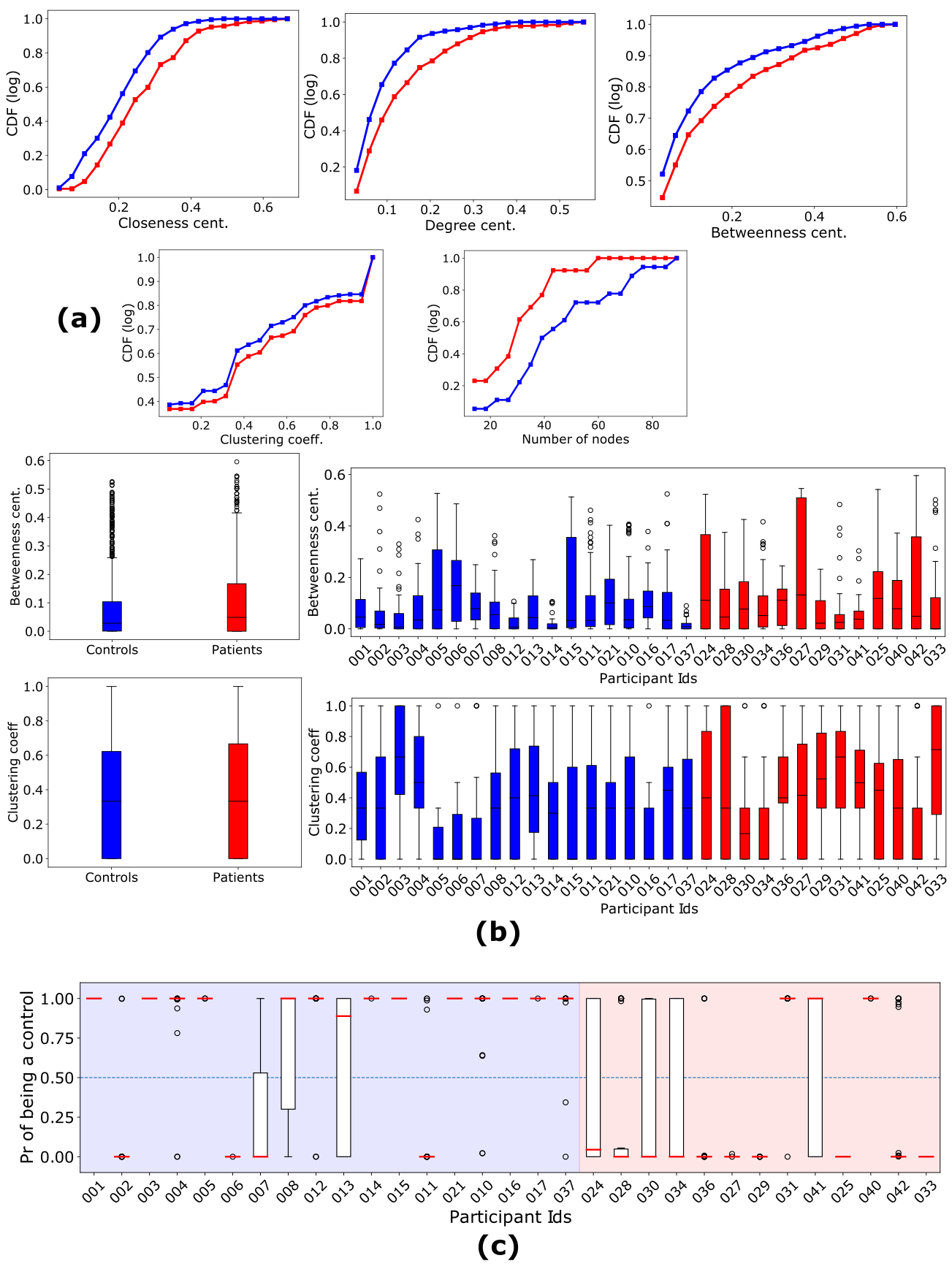


**Figure S4.** Patients and controls are represented by red and blue lines respectively. (a) Cumulative distributions of the graph-based features. Here all the patients with more than 10 nodes in the graph are considered. (b) Aggregate and individual distribution for betweenness centrality and clustering coefficient – these measures don’t produce good classification results. (c) individual classification probabilities for the best feature combination – last row in Fig 6.


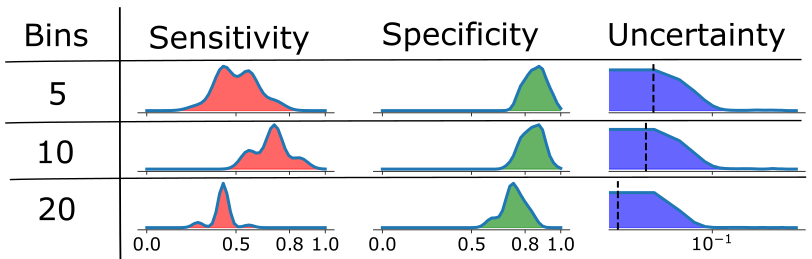


**Figure S5.** Classification performance using different number of bins in the histogram to represent the features – the setting is the same as Figure 4 with three features – segment similarity, duration of stops, and entropy.


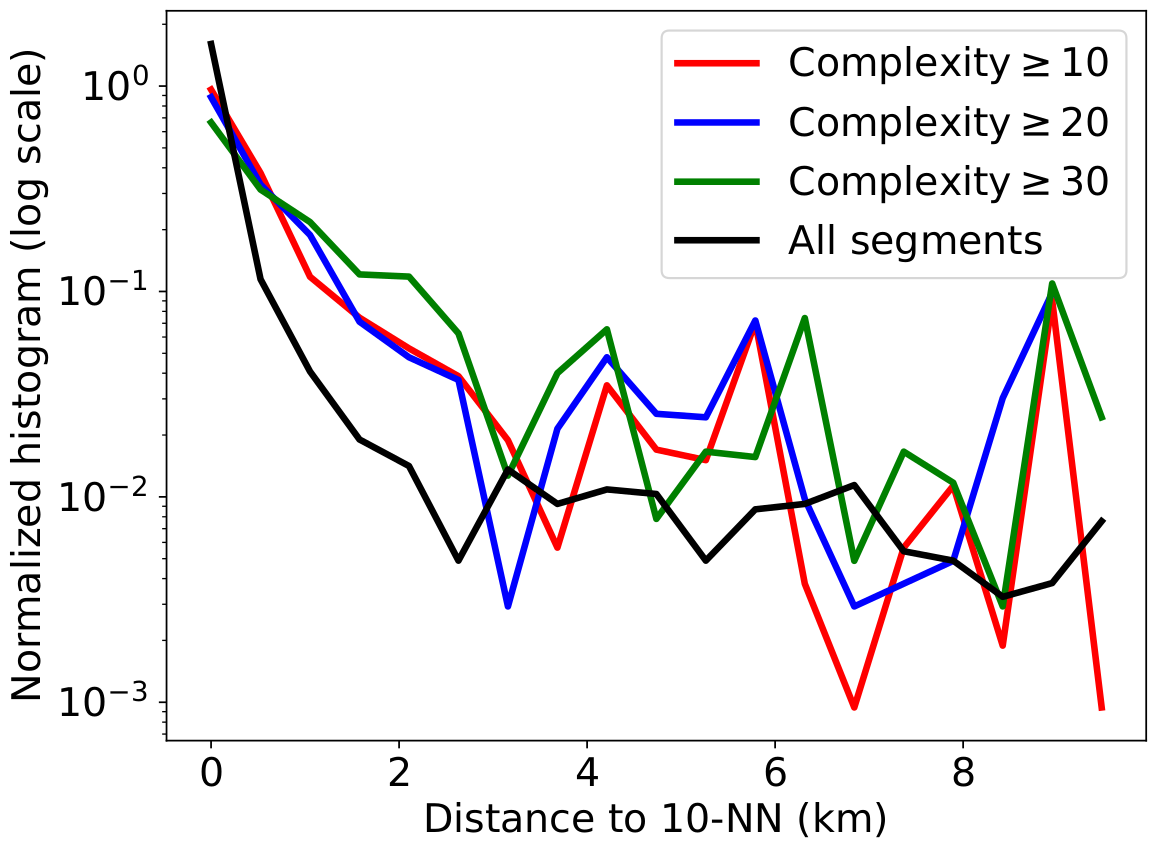


**Figure S6**. The spatial distribution of segments with different complexities. The distribution shows the distance to the 10-th nearest neighbour of segment centroids. The distances to 10-NN increase naturally with the complexity of the segments. But the distributions remain similar for different complexities. This shows that the complex segments are not concentrated in one region of the space.
